# MSCsDB: a database of single-cell transcriptomic profiles and in-depth comprehensive analyses of human mesenchymal stem cells

**DOI:** 10.1186/s40164-024-00496-5

**Published:** 2024-03-06

**Authors:** Miao Yu, Ke Sui, Zheng Wang, Xi Zhang

**Affiliations:** 1 Medical Center of Hematology; State Key Laboratory of Trauma and Chemical Poisoning；Chongqing Key Laboratory of Hematology and Microenvironment, Xinqiao Hospital of Army Medical University, Chongqing, 400037 China; 2 Bioengineering College of Chongqing University, Chongqing, 400044 China; 3 Jinfeng Laboratory, Chongqing, 401329 China; 4 Bio-Med Informatics Research Center and Clinical Research Center, The Second Affiliated Hospital, Army Medical University, Chongqing, 400037 China

**Keywords:** Database, Mesenchymal stem cells, Atlas taxonomy, Online analysis tools

## Abstract

Mesenchymal stem cells (MSCs) possess multipotent properties that make them promising candidates for immunomodulation and regenerative medicine. However, MSC heterogeneity poses challenges to their research reproducibility and clinical application. The emergence of single-cell RNA sequencing (scRNA-seq) technology has enabled a thorough examination of MSC heterogeneity, underscoring the necessity for a specialized platform to systematically analyze the published datasets derived from MSC scRNA-seq experiments. However, large-scale integration and in-depth exploration of MSC scRNA-seq datasets to comprehensively depict their developmental patterns, relationships, and knowledge are still lacking. Here, we present MSCsDB (http://mscsdb.jflab.ac.cn:18088/index/), an interactive database that has been constructed using high-quality scRNA-seq datasets from all published sources on MSCs. MSCsDB provides a one-stop interactive query for regulon activities, gene ontology enrichment, signature gene visualization and transcription factor regulon analysis. Additionally, the dedicated module within MSCsDB was developed to facilitate the evaluation of MSC quality, thereby promoting the standardization of MSC subtype usage. Notably, MSCsDB enables users to analyze their MSCs scRNA-seq data directly, yielding visually appealing outputs of exceptional quality that can be conveniently downloaded via email. Furthermore, MSCsDB integrates the current comprehensive MSC atlas taxonomy, which includes 470,000 cells and 5 tissues from 26 subjects, as publicly available references. These references provide molecular characterization and phenotypic prediction for annotating MSC subsets. In summary, MSCsDB serves as a user-friendly and contemporary data repository for human MSCs, offering a dedicated platform that enables users to effectively conduct comprehensive analyses on their individual MSCs scRNA-seq data.

**Supplementary Information:**

The online version contains supplementary material available at 10.1186/s40164-024-00496-5.

**To the Editor,**

Mesenchymal stem cells (MSCs) are multipotent cells with the capacity of self-renewal and have been studied widely as therapeutic cells for a multitude of diseases [1, 2]. The heterogeneity and disparity of MSCs significantly affects the reproducibility and consistency of experimental results and pose obstacles for clinical translation into standardized therapeutic approaches [3]. Advanced single-cell RNA sequencing (scRNA-seq) is a highly effective method for analyzing cellular heterogeneity [4, 5], while the creation of a comprehensive single-cell atlas for human MSCs remains incomplete. Furthermore, the absence of systematic utilization of unified software and parameters for standardized analysis and functional annotation of all published human MSC scRNA-seq data impedes the comparability of related research within the field [6]. To address the current limitations, we constructed MSCsDB (http://mscsdb.jflab.ac.cn:18088/index/), a comprehensive database, that depicts the connection between phenotypic characterizations and molecular signatures at single-cell resolution and provides the MSC transcriptomic landscape on the user-friendly interactive website.

MSCsDB encompasses comprehensive profiles of single-cell transcriptomes from 26 datasets across 5 healthy human tissues, systematically providing in-depth analyses across these datasets (Fig. 1A). Raw FASTQ files were downloaded and processed using Cell Ranger (v.3.0.2) and performed single-cell analysis using the Scanpy pipelines [7] (Fig. 1B). During data preprocessing steps, removal of doublets, low-quality cells, and discarded genes as well as normalization and standardization of cleaned data were performed (Fig. 1A and B). MSCsDB developed a data storage and management module, which also records manually organized meta-information includes tissue, species, sex, age, dataset ID and linked article of each dataset (Fig. 1C). Furthermore, MSCsDB developed functional modules and visualization modules, which allow users to explore phenotypic profiles, cell-type compositions and frequencies, gene signatures and their associated functions, enriched GO terms, transcriptomic-transcription factor (TF) regulatory network, and lineage trajectory inference with each interactive query (Fig. 1C). In addition, the quality assessment of MSCs is crucial for ensuring the safety and effectiveness of clinical translational therapies. With the incorporation of InferCNVpy (v0.4.2, https://github.com/icbi-lab/infercnvpy) and CopyKAT (v1.1.0) [8], MSCsDB also provides users to potentially evaluate MSC quality by analyzing copy number variations and predicting diploid/aneuploid status, which offers new methods to select the high-quality MSC subtypes for translational medicine (Fig. 1B). The construction of online database followed a front-end/back-end separation development model, utilizing Bootstrap (v5.0) and Django (v4.1.2). Additionally, a MySQL database (v5.5.21) was employed to store the database information and analysis results (Fig. 1B).

**Fig. 1:**
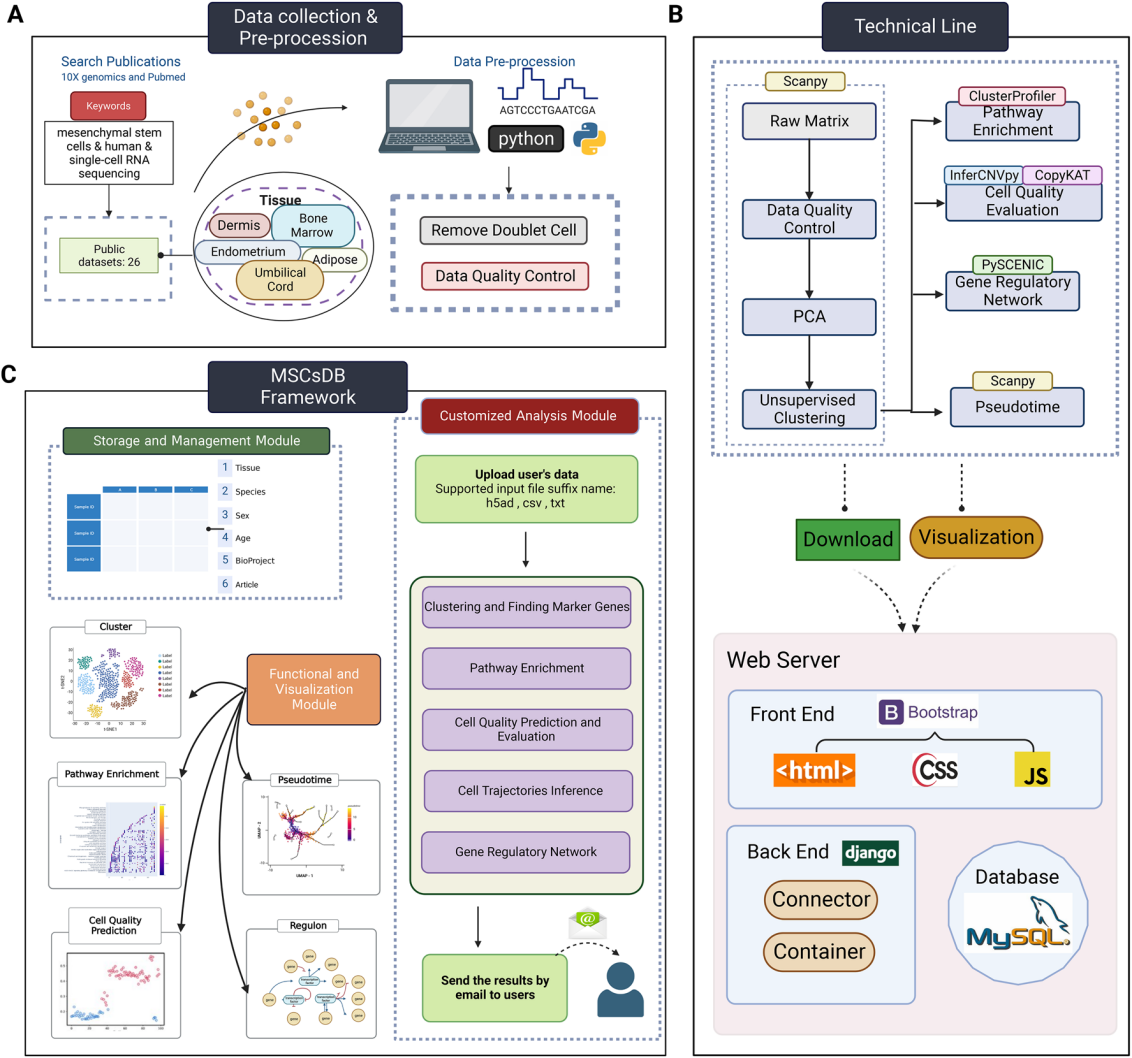
Overview of MSCsDB. **(A)** A schematic diagram showing the data collection, preprocessing and quality control steps for the MSCs datasets. **(B)** A diagram showing the technical pipeline of MSCsDB, including the data analysis workflow using various tools and the website development structure using different frameworks. **(C)** A diagram showing the seven modules of MSCsDB that provide different functions and features for users

MSCs display heterogeneity across various dimensions, encompassing variations among donors as well as disparities among tissue sources [3]. MSCsDB have successfully compiled the largest MSC atlas to date, consisting of 470,000 single-MSC transcriptomes from various tissues and donors to depict MSC heterogeneity (Fig. 1A, Additional file 1: Figure S1, S2). MSCsDB conducted a systematic delineation of each subpopulation, considering their lineage differentiation potential. Additionally, MSCsDB assessed the prospective phenotypic profiles of each subpopulation through the examination of TF regulon and their target genes, enrichment of signaling pathways, quality evaluation utilizing copy number variation analysis, and diploid/aneuploid prediction (Fig. 1B and C).

The comparability among datasets generated by different researchers is compromised due to the utilization of diverse algorithms and pipelines in public MSC single-cell transcriptome analysis. To surmount the prevailing limitations in this field, MSCsDB has devised online analysis tools that enable users to conduct comprehensive analysis of their individual data via a standardized and superior pipeline. Users can upload their data and perform quality control, data imputation, dimension reduction, clustering, pathway enrichment, quality evaluation, trajectory inference, and gene regulatory network. All steps are accompanied by adjustable parameters and plots for visualizing the analysis process. Graphs that can be visualized in PNG or editable PDF formats will be displayed and sent to the email address specified by users (Fig. 2).

**Fig. 2:**
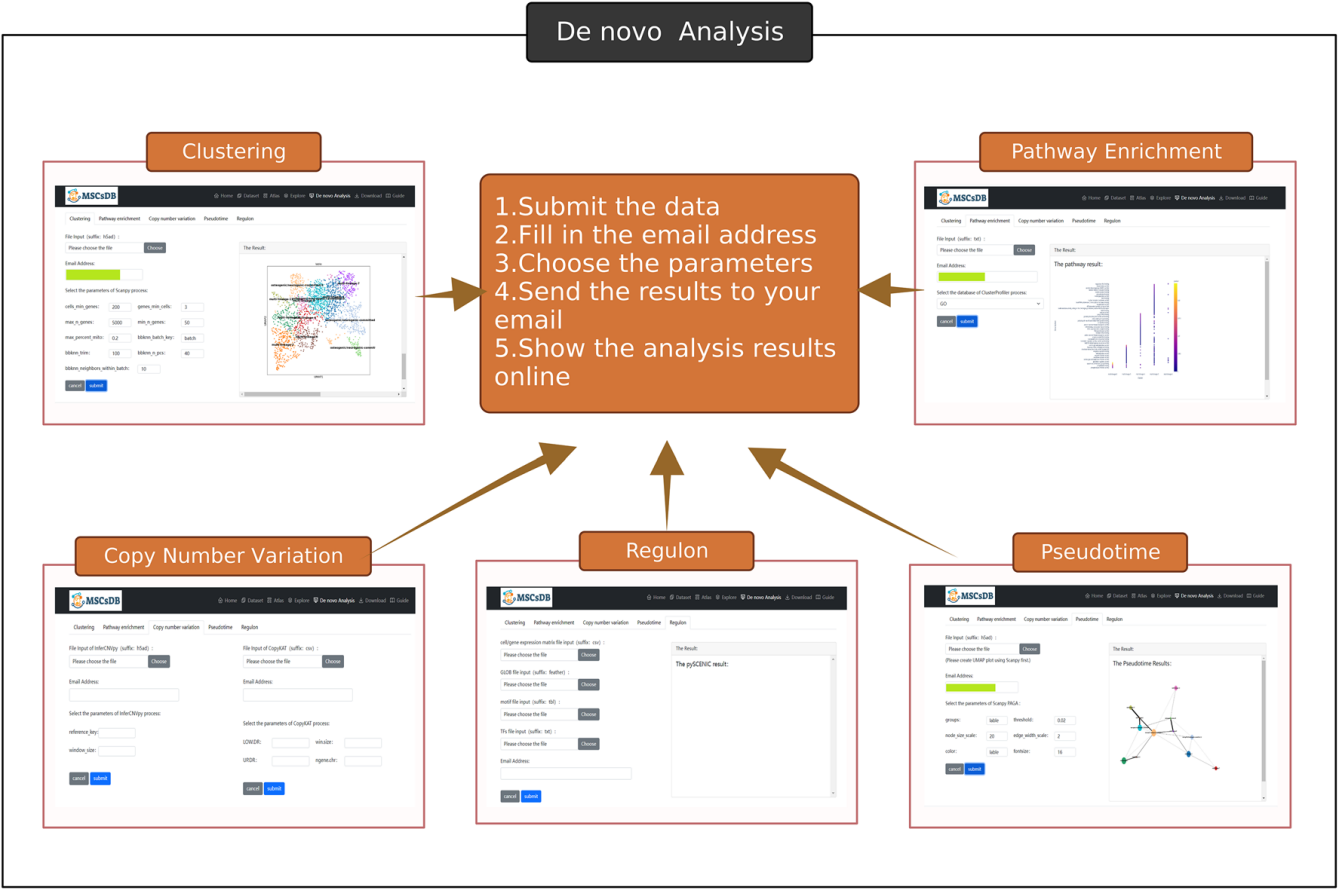
De novo analysis provided by MSCsDB. Users can submit their scRNA-seq data and run analyses for **(A)** MSC clustering and annotation using Scanpy package, **(B)** pathway enrichment using clusterprofiler package, **(C)** copy number variation and aneuploid prediction using CopyKAT and InferCNVpy packages, **(D)** Transcription factor network analysis using pyscenic package and **(E)** pseudotime trajectory inference using PAGA method. Users need to input data in a specified format, select appropriate parameters, and provide an email address to receive the analysis results. The analysis results will be sent to the users via email and displayed on the web page

To our knowledge, MSCsDB is the first dedicated data resource that aggregates the up-to-date human MSC scRNA-seq data and systematically characterizes the MSCs across various human tissues (Additional file 1: Figure S3, S4). MSCDB explores various facets encompassing molecular signatures and functional heterogeneity, as well as lineage trajectory and regulation (Additional file 1: Figure S5, S6). The utilization of analysis tools available on the MSCsDB portal empowers users to expeditiously conduct analyses on their datasets, enables comparisons between users’ datasets and those furnished by MSCsDB (Additional file 1: Figure S7, S8). MSCsDB aims to further its expansion by incorporating more tissue types, analytic tools, and omics types. This endeavor seeks to construct a comprehensive and diverse depiction of human MSC landscape at the single-cell level (Additional file 1: Table S1).

This study has some limitations. First, Due to the difficulty in determining the specific number of samples within the same group among different users and the high level of personalization, it is challenging to design a frontend interface for intra-group sample merging. However, we acknowledge that this may not be ideal for all users, and we will consider implementing a more user-friendly interface framework in future updates to address this technological challenge. Second, due to limitations in server configuration and current technological constraints, large-scale single-cell transcriptome analyses, such as analysis of millions of cells in a single-cell atlas, are currently difficult to perform directly online and to interactively display. We plan to optimize the underlying infrastructure of MSCsDB when relevant GPU-driven acceleration algorithms are well developed in this field. Third, for other single-cell multi-omics data types (such as single-cell ATAC-seq and single-cell proteomics data), there is currently limited literature reporting their application in MSC research. We will continuously update and add these data types in future database and platform upgrades (Additional file 1: Table S2).

### Supplementary Information

**Additional file1: Figure S1.** The information on MSC atlas taxonomy. (A) UMAP of all MSCs with cluster annotations, (B) UMAP of MSCs color-labelled by tissue, (C) Cell counts of MSCs from different tissues in each cluster, and (D) Cell counts of MSCs from different samples in each cluster. **Figure S2.** Differentiation scoring of MSCs on five differentiation directions. (A) Scoring of osteogenesis, chondrogenesis, adipogenesis, myogenesis and neurogenesis. (B) Scoring of representative gene expression for MSCs differentiation. **Figure S3.** Home page of MSCsDB. which includes website introduction, functionality overview, gene cloud, and website update news. **Figure S4.** Module of Dataset and link to the module of Explore. Users can view the metadata of each sample dataset, such as the original article, data repository and sequencing technology. Users can also click on the “Explore” button to view the sample’s clustering annotation, gene expression level analysis, pathway enrichment analysis, copy number variation analysis, and pseudotime analysis results. **Figure S5.** Functionality in the module of Atlas. (A) UMAP of MSCs with cluster annotations. Users can select specific clusters to view their distribution. The MSC atlas can also be classified by tissue or batch and shown separately. (B) Gene signature of MSCs. Users can analyze the cell percentage of all genes and click on the “View” button to view the gene expression levels in cells and clusters. The Gene Card database is also linked for users to view gene information. Users can also enter a specific gene in the search box to retrieve relevant information. **Figure S6.** An example of functionality in the module of Atlas. (A) Pathway enrichment analysis of MSCs from different databases. Users can switch between different databases. Users can also select specific clusters and pathways to view their enrichment status. (B) Copy number variation analysis of MSCs using copyKat and InferCNVpy packages. The copyKat software can predict whether the cells are normal cells (diploid) or tumor cells (aneuploid). The InferCNVpy package gives prediction values, so we provide chromosome heatmaps based on CNV clustering for users to distinguish between normal cells and tumor cells. (C) Pseudotime analysis of MSCs using PAGA method. We show the cell trajectory inference plot and cluster UMAP plot for a single sample. (D) Transcription factor network analysis of MSCs using pyscenic package. We provide the transcription factor network analysis result table and heatmap for a single sample’s cluster. Users can click on the “View” button in the table to view the target genes regulated by that transcription factor. **Figure S7.** De novo analysis for clustering, pathway enrichment, and quality evaluation. (A) UMAP plot of MSC clustering and annotation using Scanpy package for a sample dataset. (B) Pathway enrichment analysis using Clusterprofiler package for a sample dataset. (C) Copy number variation analysis using CopyKat and InferCNVpy packages for a sample dataset. **Figure S8.** De novo analysis for pseudotime and gene regulatory network analysis. (A) Pseudotime analysis using PAGA method for a sample dataset. (B) Gene regulatory network analysis using pyscenic package for a sample dataset. **Table S1.** Marker genes used for potency score analysis. **Table S2**. Scoring for each cluster using geneset.

## Data Availability

All data including data sources, as well as online website, are freely available at http://mscsdb.jflab.ac.cn:18088/index/, and there is no login requirement.
