# Supplementary material for: MSCsDB: a database of single-cell transcriptomic profiles and in-depth comprehensive analyses of human mesenchymal stem cells
Source: Exp Hematol Oncol. 2024 Mar 6;13:29. doi: 10.1186/s40164-024-00496-5 (PMC10919002; doi:10.1186/s40164-024-00496-5)
Supplement: Supplementary file 1 — Additional file1: Figure S1. The information on MSC atlas taxonomy. (A) UMAP of all MSCs with cluster annotations, (B) UMAP of MSCs color-labelled by tissue, (C) Cell counts of MSCs from different tissues in each cluster, and (D) Cell counts of MSCs from different samples in each cluster. Figure S2. Differentiation scoring of MSCs on five differentiation directions. (A) Scoring of osteogenesis, chondrogenesis, adipogenesis, myogenesis and neurogenesis. (B) Scoring of representative gene expression for MSCs differentiation. Figure S3. Home page of MSCsDB. which includes website introduction, functionality overview, gene cloud, and website update news. Figure S4. Module of Dataset and link to the module of Explore. Users can view the metadata of each sample dataset, such as the original article, data repository and sequencing technology. Users can also click on the “Explore” button to view the sample’s clustering annotation, gene expression level analysis, pathway enrichment analysis, copy number variation analysis, and pseudotime analysis results. Figure S5. Functionality in the module of Atlas. (A) UMAP of MSCs with cluster annotations. Users can select specific clusters to view their distribution. The MSC atlas can also be classified by tissue or batch and shown separately. (B) Gene signature of MSCs. Users can analyze the cell percentage of all genes and click on the “View” button to view the gene expression levels in cells and clusters. The Gene Card database is also linked for users to view gene information. Users can also enter a specific gene in the search box to retrieve relevant information. Figure S6. An example of functionality in the module of Atlas. (A) Pathway enrichment analysis of MSCs from different databases. Users can switch between different databases. Users can also select specific clusters and pathways to view their enrichment status. (B) Copy number variation analysis of MSCs using copyKat and InferCNVpy packages. The copyKat software can predi [file 40164_2024_496_MOESM1_ESM.zip › Additional file/Figure S3.pdf]

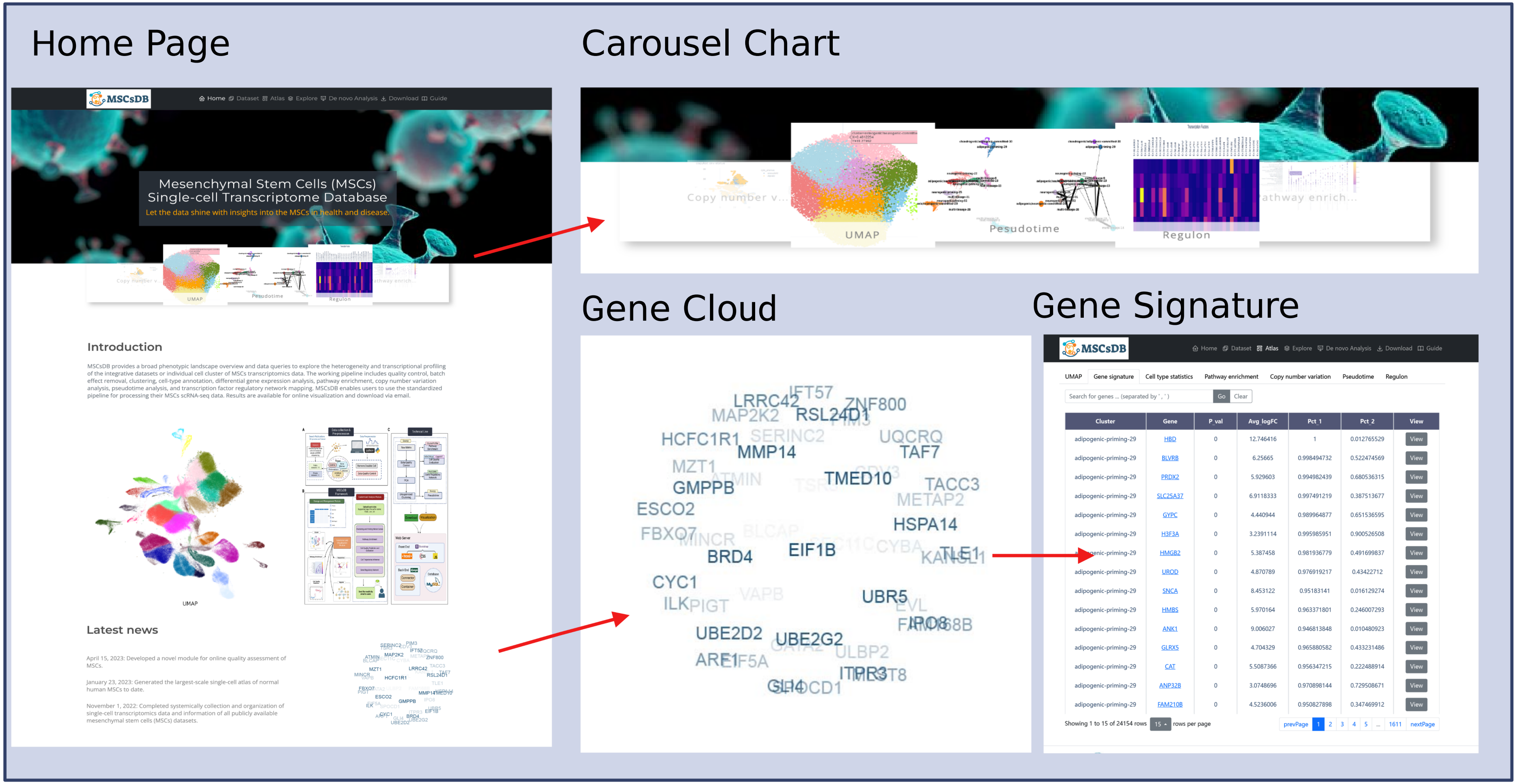

Home Page
Carousel Chart
Gene Cloud
Gene Signature
MSCsDB
Home Dataset Atlas Explore De novo Analysis Download Guide
Mesenchymal Stem Cells (MSCs)
Single-cell Transcriptome Database
Let the data shine with insights into the MSCs in health and disease.
Copy number v...
UMAP
Pesudotime
Regulon
athway enrich...
Introduction
MSCsDB provides a broad phenotypic landscape overview and data queries to explore the heterogeneity and transcriptional profiling of the integrative datasets or individual cell cluster of MSCs transcriptomics data. The working pipeline includes quality control, batch effect removal, clustering, cell-type annotation, differential gene expression analysis, pathway enrichment, copy number variation analysis, pseudotime analysis, and transcription factor regulatory network mapping. MSCsDB enables users to use the standardized pipeline for processing their MSCs scRNA-seq data. Results are available for online visualization and download via email.
UMAP
Latest news
April 15, 2023: Developed a novel module for online quality assessment of MSCs.
January 23, 2023: Generated the largest-scale single-cell atlas of normal human MSCs to date.
November 1, 2022: Completed systemically collection and organization of single-cell transcriptomics data and information of all publicly available mesenchymal stem cells (MSCs) datasets.
LRRC42
IFT57
ZNF800
MAP2K2
RSL24D1
HCFC1R1
SERINC2
UQCRQ
MMP14
TAF7
MZT1
GMPPB
TMED10
TACC3
METAP2
ESCO2
HSPA14
FBXO7
BRD4
EIF1B
CYBA
CYC1
ILK
PIGT
VAPB
UBR5
UBE2D2
UBE2G2
ULBP2
ITPR3
UMAP Gene signature Cell type statistics Pathway enrichment Copy number variation Pseudotime Regulon
Search for genes ... (separated by ' , ')
Go
Clear
Cluster | Gene | P val | Avg logFC | Pct 1 | Pct 2 | View
adipogenic-priming-29 | HBD | 0 | 12.746416 | 1 | 0.012765529 | View
adipogenic-priming-29 | BLVRB | 0 | 6.25665 | 0.998494732 | 0.522474569 | View
adipogenic-priming-29 | PRDX2 | 0 | 5.929603 | 0.994982439 | 0.680536315 | View
adipogenic-priming-29 | SLC25A37 | 0 | 6.9118333 | 0.997491219 | 0.387513677 | View
adipogenic-priming-29 | GYPC | 0 | 4.440944 | 0.989964877 | 0.651536595 | View
adipogenic-priming-29 | H3F3A | 0 | 3.2391114 | 0.995985951 | 0.900526508 | View
adipogenic-priming-29 | HMGB2 | 0 | 5.387458 | 0.981936779 | 0.491699837 | View
adipogenic-priming-29 | UROD | 0 | 4.870789 | 0.976919217 | 0.43422712 | View
adipogenic-priming-29 | SNCA | 0 | 8.453122 | 0.95183141 | 0.016129274 | View
adipogenic-priming-29 | HMBS | 0 | 5.970164 | 0.963371801 | 0.246007293 | View
adipogenic-priming-29 | ANK1 | 0 | 9.006027 | 0.946813848 | 0.010480923 | View
adipogenic-priming-29 | GLRX5 | 0 | 4.704329 | 0.965880582 | 0.433231486 | View
adipogenic-priming-29 | CAT | 0 | 5.5087366 | 0.956347215 | 0.222488914 | View
adipogenic-priming-29 | ANP32B | 0 | 3.0748696 | 0.970898144 | 0.729508671 | View
adipogenic-priming-29 | FAM210B | 0 | 4.5236006 | 0.950827898 | 0.347469912 | View
Showing 1 to 15 of 24154 rows 15 rows per page
prevPage 1 2 3 4 5 ... 1611 nextPage
