# Supplementary material for: MSCsDB: a database of single-cell transcriptomic profiles and in-depth comprehensive analyses of human mesenchymal stem cells
Source: Exp Hematol Oncol. 2024 Mar 6;13:29. doi: 10.1186/s40164-024-00496-5 (PMC10919002; doi:10.1186/s40164-024-00496-5)
Supplement: Supplementary file 1 — Additional file1: Figure S1. The information on MSC atlas taxonomy. (A) UMAP of all MSCs with cluster annotations, (B) UMAP of MSCs color-labelled by tissue, (C) Cell counts of MSCs from different tissues in each cluster, and (D) Cell counts of MSCs from different samples in each cluster. Figure S2. Differentiation scoring of MSCs on five differentiation directions. (A) Scoring of osteogenesis, chondrogenesis, adipogenesis, myogenesis and neurogenesis. (B) Scoring of representative gene expression for MSCs differentiation. Figure S3. Home page of MSCsDB. which includes website introduction, functionality overview, gene cloud, and website update news. Figure S4. Module of Dataset and link to the module of Explore. Users can view the metadata of each sample dataset, such as the original article, data repository and sequencing technology. Users can also click on the “Explore” button to view the sample’s clustering annotation, gene expression level analysis, pathway enrichment analysis, copy number variation analysis, and pseudotime analysis results. Figure S5. Functionality in the module of Atlas. (A) UMAP of MSCs with cluster annotations. Users can select specific clusters to view their distribution. The MSC atlas can also be classified by tissue or batch and shown separately. (B) Gene signature of MSCs. Users can analyze the cell percentage of all genes and click on the “View” button to view the gene expression levels in cells and clusters. The Gene Card database is also linked for users to view gene information. Users can also enter a specific gene in the search box to retrieve relevant information. Figure S6. An example of functionality in the module of Atlas. (A) Pathway enrichment analysis of MSCs from different databases. Users can switch between different databases. Users can also select specific clusters and pathways to view their enrichment status. (B) Copy number variation analysis of MSCs using copyKat and InferCNVpy packages. The copyKat software can predi [file 40164_2024_496_MOESM1_ESM.zip › Additional file/Figure S4.pdf]

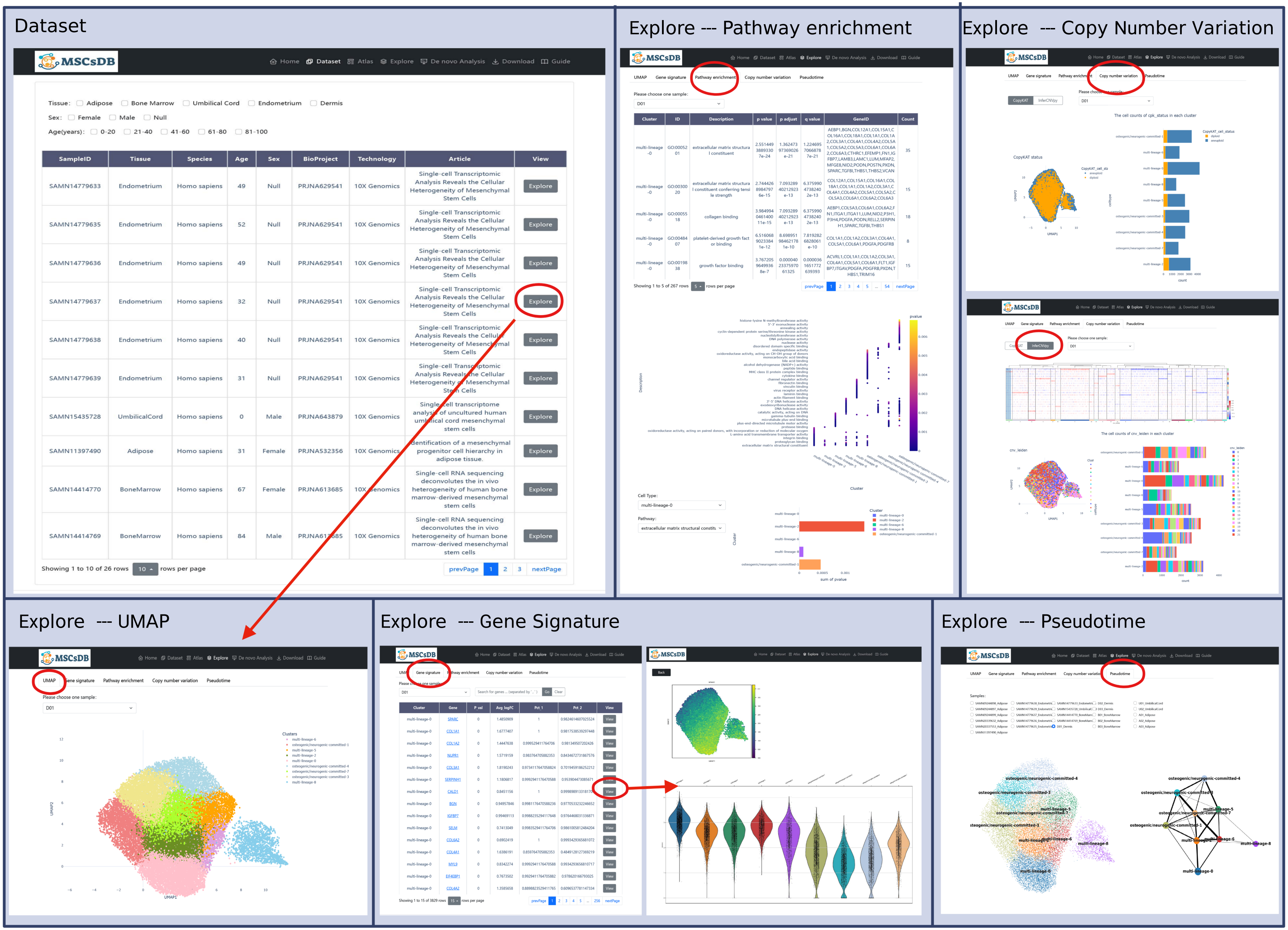
Dataset
Explore --- Pathway enrichment
Explore --- Copy Number Variation
Explore --- UMAP
Explore --- Gene Signature
Explore --- Pseudotime
MSCsDB
Home
Dataset
Atlas
Explore
De novo Analysis
Download
Guide
Tissue: Adipose Bone Marrow Umbilical Cord Endometrium Dermis
Sex: Female Male Null
Age(years): 0-20 21-40 41-60 61-80 81-100
SampleID
Tissue
Species
Age
Sex
BioProject
Technology
Article
View
SAMN14779633 Endometrium Homo sapiens 49 Null PRJNA629541 10X Genomics Single-cell Transcriptomic Analysis Reveals the Cellular Heterogeneity of Mesenchymal Stem Cells Explore
SAMN14779635 Endometrium Homo sapiens 52 Null PRJNA629541 10X Genomics Single-cell Transcriptomic Analysis Reveals the Cellular Heterogeneity of Mesenchymal Stem Cells Explore
SAMN14779636 Endometrium Homo sapiens 49 Null PRJNA629541 10X Genomics Single-cell Transcriptomic Analysis Reveals the Cellular Heterogeneity of Mesenchymal Stem Cells Explore
SAMN14779637 Endometrium Homo sapiens 32 Null PRJNA629541 10X Genomics Single-cell Transcriptomic Analysis Reveals the Cellular Heterogeneity of Mesenchymal Stem Cells Explore
SAMN14779638 Endometrium Homo sapiens 40 Null PRJNA629541 10X Genomics Single-cell Transcriptomic Analysis Reveals the Cellular Heterogeneity of Mesenchymal Stem Cells Explore
SAMN14779639 Endometrium Homo sapiens 31 Null PRJNA629541 10X Genomics Single-cell Transcriptomic Analysis Reveals the Cellular Heterogeneity of Mesenchymal Stem Cells Explore
SAMN15435728 UmbilicalCord Homo sapiens 0 Male PRJNA643879 10X Genomics Single cell transcriptome analysis of uncultured human umbilical cord mesenchymal stem cells Explore
SAMN11397490 Adipose Homo sapiens 31 Female PRJNA532356 10X Genomics Identification of a mesenchymal progenitor cell hierarchy in adipose tissue. Explore
SAMN14414770 BoneMarrow Homo sapiens 67 Female PRJNA613685 10X Genomics Single-cell RNA sequencing deconvolutes the in vivo heterogeneity of human bone marrow-derived mesenchymal stem cells Explore
SAMN14414769 BoneMarrow Homo sapiens 84 Male PRJNA613685 10X Genomics Single-cell RNA sequencing deconvolutes the in vivo heterogeneity of human bone marrow-derived mesenchymal stem cells Explore
Showing 1 to 10 of 26 rows 10 rows per page
prevPage 1 2 3 nextPage
UMAP
Gene signature
Pathway enrichment
Copy number variation
Pseudotime
Please choose one sample:
D01
