# Supplementary material for: MSCsDB: a database of single-cell transcriptomic profiles and in-depth comprehensive analyses of human mesenchymal stem cells
Source: Exp Hematol Oncol. 2024 Mar 6;13:29. doi: 10.1186/s40164-024-00496-5 (PMC10919002; doi:10.1186/s40164-024-00496-5)
Supplement: Supplementary file 1 — Additional file1: Figure S1. The information on MSC atlas taxonomy. (A) UMAP of all MSCs with cluster annotations, (B) UMAP of MSCs color-labelled by tissue, (C) Cell counts of MSCs from different tissues in each cluster, and (D) Cell counts of MSCs from different samples in each cluster. Figure S2. Differentiation scoring of MSCs on five differentiation directions. (A) Scoring of osteogenesis, chondrogenesis, adipogenesis, myogenesis and neurogenesis. (B) Scoring of representative gene expression for MSCs differentiation. Figure S3. Home page of MSCsDB. which includes website introduction, functionality overview, gene cloud, and website update news. Figure S4. Module of Dataset and link to the module of Explore. Users can view the metadata of each sample dataset, such as the original article, data repository and sequencing technology. Users can also click on the “Explore” button to view the sample’s clustering annotation, gene expression level analysis, pathway enrichment analysis, copy number variation analysis, and pseudotime analysis results. Figure S5. Functionality in the module of Atlas. (A) UMAP of MSCs with cluster annotations. Users can select specific clusters to view their distribution. The MSC atlas can also be classified by tissue or batch and shown separately. (B) Gene signature of MSCs. Users can analyze the cell percentage of all genes and click on the “View” button to view the gene expression levels in cells and clusters. The Gene Card database is also linked for users to view gene information. Users can also enter a specific gene in the search box to retrieve relevant information. Figure S6. An example of functionality in the module of Atlas. (A) Pathway enrichment analysis of MSCs from different databases. Users can switch between different databases. Users can also select specific clusters and pathways to view their enrichment status. (B) Copy number variation analysis of MSCs using copyKat and InferCNVpy packages. The copyKat software can predi [file 40164_2024_496_MOESM1_ESM.zip › Additional file/Figure S5.pdf]

A

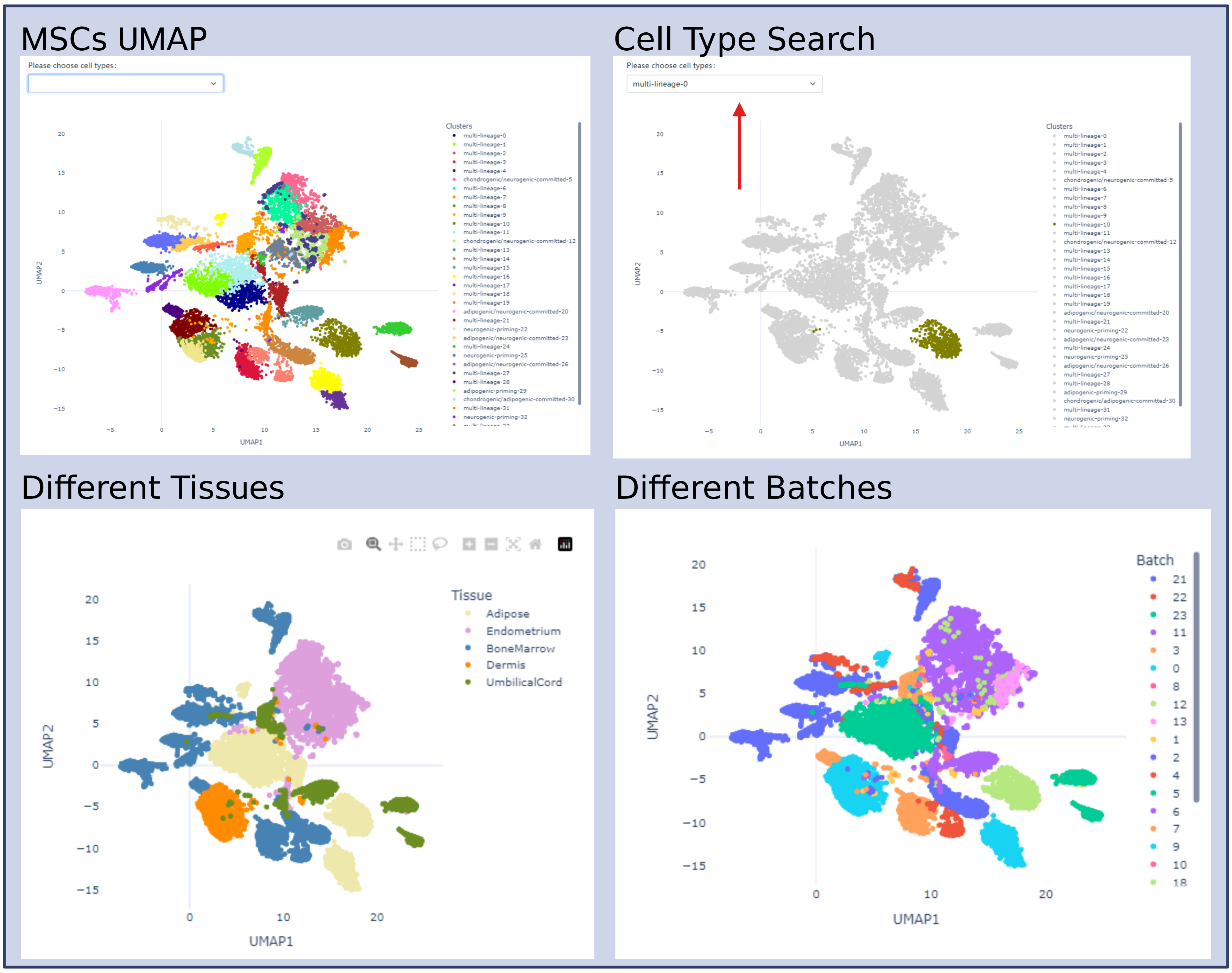
MSCs UMAP
Please choose cell types:
UMAP1
UMAP2
Cell Type Search
Please choose cell types:
multi-lineage-0
Clusters
Different Tissues
Tissue
Adipose
Endometrium
BoneMarrow
Dermis
UmbilicalCord
UMAP1
UMAP2
Different Batches
Batch
21
22
23
11
3
0
8
12
13
1
2
4
5
6
7
9
10
18
UMAP1
UMAP2

B

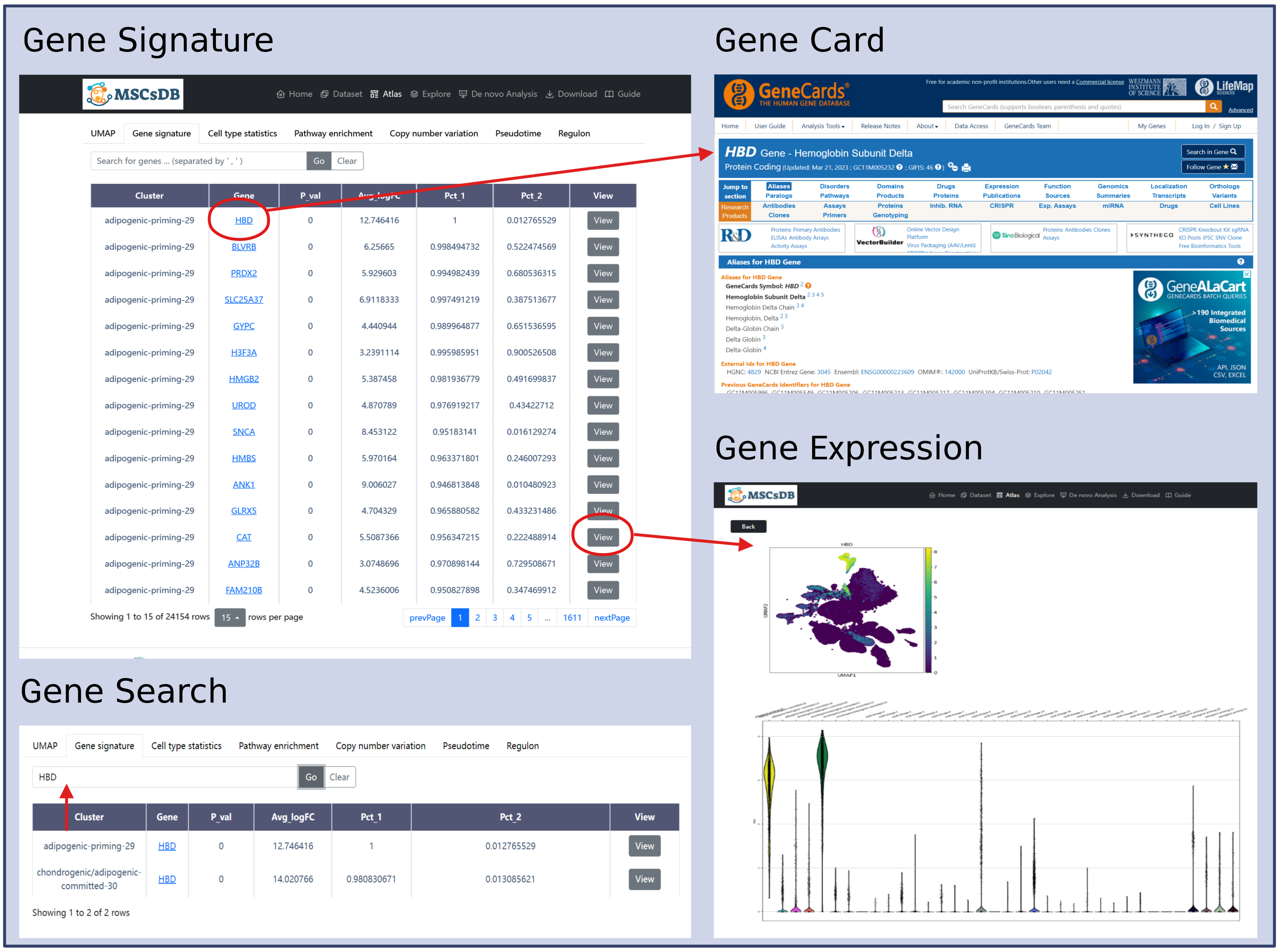
Gene Signature
MSCsDB
Home
Dataset
Atlas
Explore
De novo Analysis
Download
Guide
UMAP
Gene signature
Cell type statistics
Pathway enrichment
Copy number variation
Pseudotime
Regulon
Search for genes ... (separated by ' , ')
Go
Clear
Cluster
Gene
P_val
Pct_1
Pct_2
View
adipogenic-priming-29
HBD
BLVRB
PRDX2
SLC25A37
GYPC
H3F3A
HMGB2
UROD
SNCA
HMBS
ANK1
GLRX5
CAT
ANP32B
FAM210B
Showing 1 to 15 of 24154 rows
rows per page
prevPage
nextPage
Gene Card
GeneCards
THE HUMAN GENE DATABASE
LifeMap
HBD Gene - Hemoglobin Subunit Delta
Protein Coding
Aliases for HBD Gene
GeneALaCart
Gene Expression
MSCsDB
Back
Gene Search
UMAP
Gene signature
Cell type statistics
Pathway enrichment
Copy number variation
Pseudotime
Regulon
HBD
Go
Clear
Cluster
Gene
P_val
Avg_logFC
Pct_1
Pct_2
View
adipogenic-priming-29
HBD
0
12.746416
1
0.012765529
chondrogenic/adipogenic-committed-30
HBD
0
14.020766
0.980830671
0.013085621
Showing 1 to 2 of 2 rows
