# Supplementary material for: MSCsDB: a database of single-cell transcriptomic profiles and in-depth comprehensive analyses of human mesenchymal stem cells
Source: Exp Hematol Oncol. 2024 Mar 6;13:29. doi: 10.1186/s40164-024-00496-5 (PMC10919002; doi:10.1186/s40164-024-00496-5)
Supplement: Supplementary file 1 — Additional file1: Figure S1. The information on MSC atlas taxonomy. (A) UMAP of all MSCs with cluster annotations, (B) UMAP of MSCs color-labelled by tissue, (C) Cell counts of MSCs from different tissues in each cluster, and (D) Cell counts of MSCs from different samples in each cluster. Figure S2. Differentiation scoring of MSCs on five differentiation directions. (A) Scoring of osteogenesis, chondrogenesis, adipogenesis, myogenesis and neurogenesis. (B) Scoring of representative gene expression for MSCs differentiation. Figure S3. Home page of MSCsDB. which includes website introduction, functionality overview, gene cloud, and website update news. Figure S4. Module of Dataset and link to the module of Explore. Users can view the metadata of each sample dataset, such as the original article, data repository and sequencing technology. Users can also click on the “Explore” button to view the sample’s clustering annotation, gene expression level analysis, pathway enrichment analysis, copy number variation analysis, and pseudotime analysis results. Figure S5. Functionality in the module of Atlas. (A) UMAP of MSCs with cluster annotations. Users can select specific clusters to view their distribution. The MSC atlas can also be classified by tissue or batch and shown separately. (B) Gene signature of MSCs. Users can analyze the cell percentage of all genes and click on the “View” button to view the gene expression levels in cells and clusters. The Gene Card database is also linked for users to view gene information. Users can also enter a specific gene in the search box to retrieve relevant information. Figure S6. An example of functionality in the module of Atlas. (A) Pathway enrichment analysis of MSCs from different databases. Users can switch between different databases. Users can also select specific clusters and pathways to view their enrichment status. (B) Copy number variation analysis of MSCs using copyKat and InferCNVpy packages. The copyKat software can predi [file 40164_2024_496_MOESM1_ESM.zip › Additional file/Figure S7.pdf]

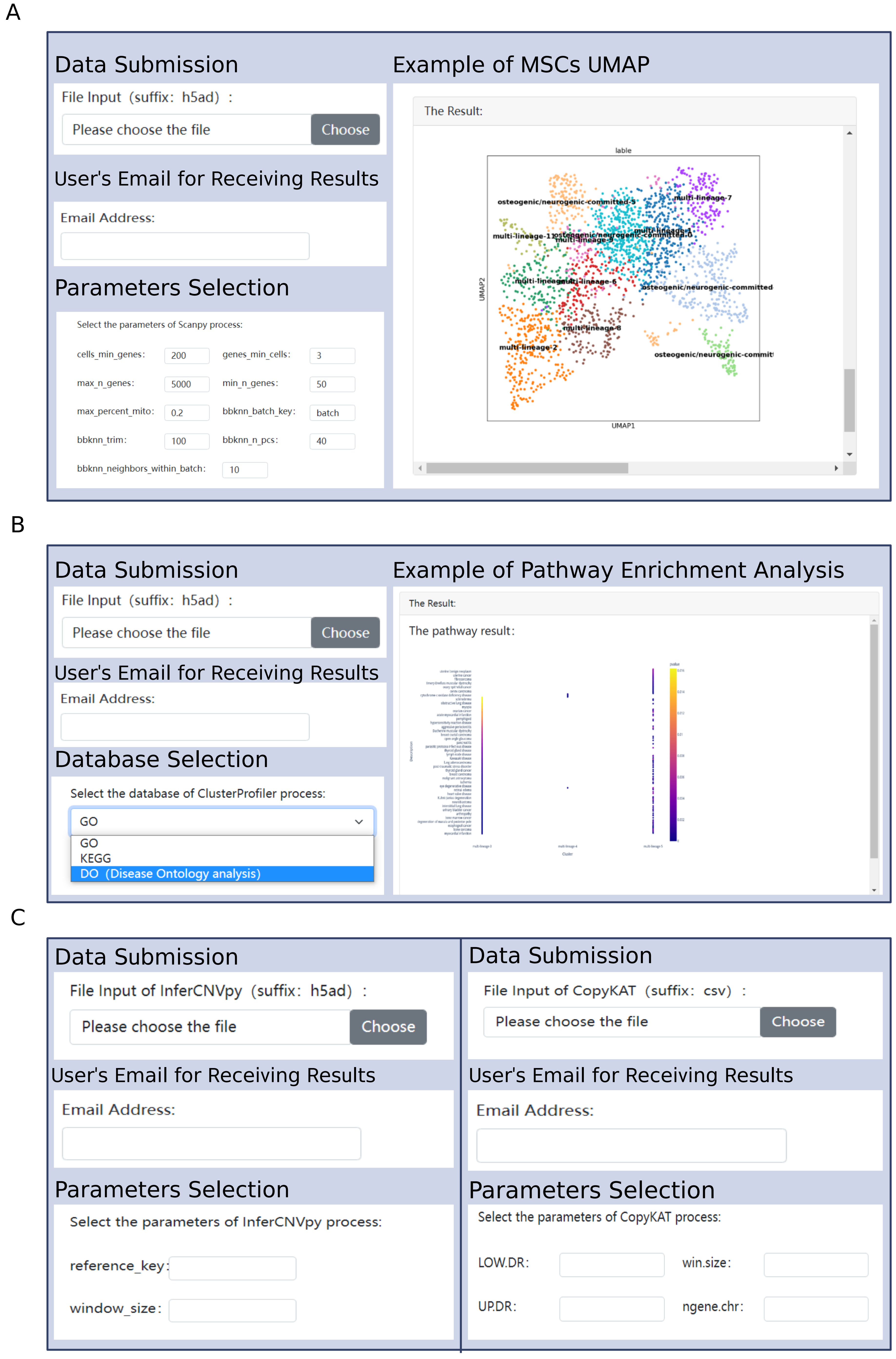
A
Data Submission
File Input（suffix：h5ad）：
Please choose the file
Choose
User's Email for Receiving Results
Email Address:
Parameters Selection
Select the parameters of Scanpy process:
cells_min_genes: 200
genes_min_cells: 3
max_n_genes: 5000
min_n_genes: 50
max_percent_mito: 0.2
bbknn_batch_key: batch
bbknn_trim: 100
bbknn_n_pcs: 40
bbknn_neighbors_within_batch: 10
Example of MSCs UMAP
The Result:
lable
osteogenic/neurogenic-committed-5
multi-lineage-7
multi-lineage-1
multi-lineage-9
multi-lineage-6
osteogenic/neurogenic-committed
multi-lineage-8
multi-lineage-2
osteogenic/neurogenic-commit
UMAP2
UMAP1
B
Data Submission
File Input（suffix：h5ad）：
Please choose the file
Choose
User's Email for Receiving Results
Email Address:
Database Selection
Select the database of ClusterProfiler process:
GO
GO
KEGG
DO（Disease Ontology analysis）
Example of Pathway Enrichment Analysis
The Result:
The pathway result:
C
Data Submission
File Input of InferCNVpy（suffix：h5ad）：
Please choose the file
Choose
User's Email for Receiving Results
Email Address:
Parameters Selection
Select the parameters of InferCNVpy process:
reference_key:
window_size:
Data Submission
File Input of CopyKAT（suffix：csv）：
Please choose the file
Choose
User's Email for Receiving Results
Email Address:
Parameters Selection
Select the parameters of CopyKAT process:
LOW.DR:
win.size:
UP.DR:
ngene.chr:
